# A case report of appendix mucinous adenocarcinoma that recurred after additional surgery and a brief literature review

**DOI:** 10.1186/s12893-020-00842-4

**Published:** 2020-08-10

**Authors:** Wei Chen, Jun-Wen Ye, Xiao-ping Tan, Xiang Peng, Yan Zhang, Jing-Lin Liang, Mei-Jin Huang

**Affiliations:** 1 Department of Colorectal Surgery, the Sixth Affiliated Hospital, Sun Yat-sen University, Guangzhou, Guangdong 510655 People’s Republic of China; 2 Guangdong Provincial Key Laboratory of Colorectal and Pelvic Floor Disease, The Sixth Affiliated Hospital of Sun Yat-sen University, Guangzhou, 510655 China; 3 Guangdong Research Institute of Gastroenterology, The Sixth Affiliated Hospital of Sun Yat-sen University, Guangzhou, 510655 China; 4 Department of Emergency, The Second Affiliated Hospital of Guangzhou Medical University, Guangzhou, 510655 China; 5 Department of Gastroenterology, the Sixth Affiliated Hospital, Sun Yat-sen University, Guangzhou, 510655 China; 6 Department of Medicine Oncology, the Sixth Affiliated Hospital, Sun Yat-sen University, Guangzhou, 510655 China

**Keywords:** Colorectal cancer, Appendiceal mucinous adenocarcinoma, HIPEC, Recurrence

## Abstract

**Background:**

The clinical incidence of appendiceal mucinous adenocarcinoma is low. Moreover, the case reports of postoperative relapse after surgery are rarely based on literature search results. Here, we report such a case spanning nearly 7 years and and review the relevant literature.

**Case presentation:**

A 50-year-old female underwent additional surgery after appendectomy, and pathological examination confirmed mucinous adenocarcinoma. The patients underwent HIPEC (hyperthermic intraoperative chemotherapy) and adjuvant chemotherapy. Twenty-six months after the previous surgeries, another surgery, HIPEC, and adjuvant chemotherapy were performed again due to tumour recurrence. To date, the follow-up time is 43 months, and no recurrence or metastasis has been found.

**Conclusions:**

Appendix mucinous adenocarcinoma has a poor prognosis and the diagnosis depends on pathological and immunohistochemical examinations. Its clinical manifestations are non-specific, and CRS + HIPEC should be used for treatment, which is safe and effective.

## Background

Appendiceal adenocarcinoma accounts for < 0.5% of all gastrointestinal neoplasms. it is extremely difficult to diagnose prior to surgical inspection, and it usually depends on the pathology following appendectomy [1]. Due to the small sample size and range of clinical presentations, the diagnosis of appendiceal carcinoma remains challenging for physicians. Although some literatures associated with mucinous adenocarcinoma of the appendix have been reported, there are few reports about its treatment after relapse based on literature search results [2–5]. Here, we report such a case, in which the patient underwent the surgery three times associated with the appendiceal adenocarcinoma and review the relevant literature.

## Case presentation

**Fig. 1:**
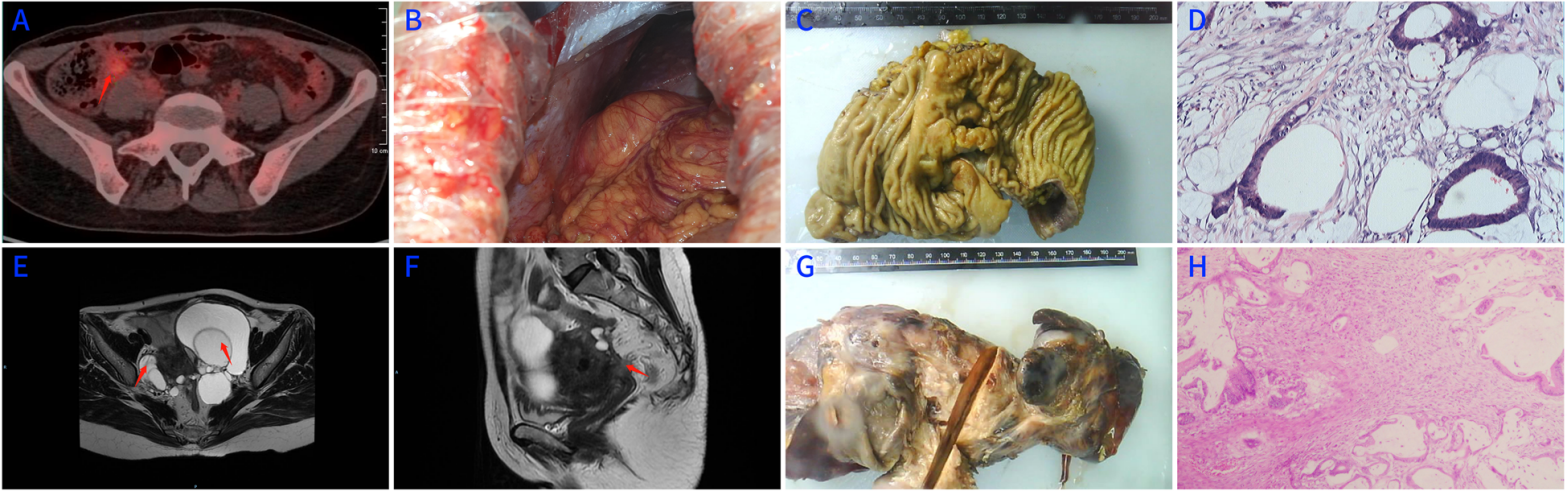
**a** The results of PET showed that the intestinal soft tissues in the ileocecal area are slightly parametrized and the metabolism is slightly active. **b** The pictures of ileocecal part from open surgery in September 11, 2013. **c** The surgically resected specimen. **d** Histopathological examination of colon, the cavity is filled with mucus like mucus cyst. **e**, **f** Pelvic MRI showing multiple cystic lesions in the bilateral attachment area (arrows). **g** The surgically resected specimen in November 12, 2015. **h** Histopathological examination of surgically resected specimen, the cavity is filled with mucus like mucus cyst

The patient was 50 years old, and she underwent appendectomy + laparoscopic removal of an ovarian cyst + peritonectomy in the external hospital on August 29, 2013, due to “right lower abdominal pain for 3 days”. The postoperative pathology was as follows: (1) mucinous adenocarcinoma of the appendix; (2) luteal haematoma of the ovary; and (3) metastatic mucinous adenocarcinoma nodule of the peritoneum. On September 6, 2013, she was transferred to our hospital. The PET results showed that the intestinal soft tissues in the ileocecal area were slightly parametrized and that metabolism was slightly active (Fig. 1a). Additional surgery was performed on September 11, 2013, and the surgical method was ileocecal resection + small intestinal mesenteric nodule biopsy + HIPEC (Fig. 1b). The postoperative pathology results were as follows: (1) (mesenteric lymph nodes) 8 lymph nodes, no cancer metastasis (0/8), and 4 cancer nodes; (2) (mesenteric nodule) under the microscope, there were 5 cancer nodules; (3) (colon tumour resection specimen) local erosion of the colon mucosa, infiltration of mucinous adenocarcinoma in the serous layer, infiltration of a large number of inflammatory cells, with multinucleated giant cell reaction, no tumour thrombus in the vasculature, no cancer infiltration in the nerve bundle, and no cancer involvement in two incisional limbuses; (4) immune group: Ki-67 approximately 10% of cells (+), MH (+), MSH2 (+), MSHB (+), PMS2 (+), CDX2 (+), and HER2 (−) (Fig. 1c, d). Regular chemotherapy was used 12 times after surgery, and the adopted regimen was oxaliplatin + 5-FU.

The patient regularly returned to the hospital for a full physical examination after surgery, and the examination revealed tumour recurrence at 26 months after surgery. On November 12, 2015, the results of pelvic MRI showed that multiple cystic lesions were found in the bilateral attachment area (Fig. 1e, f). On July 13, 2016, rectal metastasis resection + small intestine partial resection + hysterectomy + bilateral fallopian tube + ovariectomy + HIPEC were performed in open surgery under general anaesthesia. After the operation, regular chemotherapy was performed 8 times, and the adopted regimen was FOLFIRI.

Under general anaesthesia, a laparotomy was performed. During the operation, bilateral attachments were found to be cystic solid tumours, which were fixed to some of the small intestine and rectum, with mucus adhered around and enlarged lymph nodes around the intestine. Abdominal wall mucus was taken intraoperatively for rapid pathology, suggesting that mucus glands were visible in the mucus tissue and the glands with moderate atypia. Rectal metastasis resection + intestinal adhesion lysis + partial intestinal resection + hysterectomy + bilateral fallopian tube + ovariectomy + HIPEC were performed according to the exploration and rapid pathological results. Pipe stapler anastomosis was used in radical operation. The patient recovered smoothly after the operation.

The postoperative pathology was as follows: (rectal specimen) intestinal mucosal infiltration of mucosal adenocarcinoma can be seen at the full thickness of the intestine, with negative margins, and another cancer nodule was present in the mesenteric lymph nodes (0/6); (uterus + double attachments) cervical and bilateral attachments can be found with mucinous adenocarcinoma invasion, and the immunohistochemical results were as follows: CK7 (+), CK20 (+), and CDX2 (+); the results support the origin of the digestive tract (Fig. 1g, h). The patient returned to the hospital for regular review after the operation. To date, the follow-up time is 43 months, and no recurrence or metastasis has been found.

## Discussion and conclusions

Appendiceal neoplasms belong to a group of neoplasms with a lower incidence rate among all digestive tract tumours. In all specimens after appendectomy, the tumours account for approximately 0.7 to 1.4%, of which the incidence of appendix mucinous adenocarcinoma accounts for 0.01% ~ 0.08% [6–8]. Therefore, the rate of misdiagnosis is high. The peak age of onset is between 50 and 60 years old, and most patients are female; its occurrence may be related to long-term inflammation and infiltration of the appendix.

Tumour markers of appendiceal mucinous adenocarcinoma can be elevated, such as CA125, CA199 and CEA. Preoperative tumour markers are more consistent with TNM staging [9]. Postoperative tumour markers in patients are positively associated with poor prognosis. Moreover, imaging examination is an important detection method in the diagnosis of appendix mucinous adenocarcinoma. Colonoscopy can be used to biopsy the diseased part, and the diagnosis can be confirmed by pathological examination. MRI and PET-CT combine the metabolic state of the lesion with the anatomical structure, which is helpful for improving the detection rate of the tumour and guiding the development of the diagnosis and treatment plan [10–12].

In any case, at the time of the first operation, the appendix and the adipose tissue around its mesentery should be removed. If any liquid or mucus is found, cytological examination should be performed [13]. Because mucinous adenocarcinoma is prone to intracavitary implantation, care should be taken to protect the surgical field and incision during surgery to avoid breaking the tumour and the surgical field should be repeatedly washed with 0.5% 5-FU solution [14]. In our case, the tumour was positive at the incisal margin of the appendix, and the CEA level increased slightly before surgery. Combined with the PET examination results and after consultation with the patient’s family, we decided to perform ileocecal resection + HIPEC, followed by regular chemotherapy after surgery. If negative results are obtained from the interoperative frozen sections of lymph nodes in and along the appendiceal artery, then right hemicolectomy can be avoided. In addition, a negative incision margin for the appendix can be obtained by simple appendectomy, which can also preserve the function of the ascending colon and the ileocecal valve [15]. If a negative margin is not obtained, ileocecal excision can be performed.

It is recommended that women with menopausal appendix mucinous adenocarcinoma undergo menopause before the tumour is removed together with the ovary, which can prevent metastasis and improve the survival rate [16]. As shown in our case, we followed up with the patient, but on the MRI examination 23 months after the right hemicolectomy, a mass was found in the uterus, bilateral ovaries and fallopian tubes, and there was a partially calcified wall. We considered that the tumour recurred, which was confirmed in the subsequent surgery. According to the histological report, a tumour with the same characteristics as the previous one was found in the uterus, bilateral ovaries and fallopian tubes. IFCCs are the pathological basis of recurrence and metastasis of gastrointestinal tract malignant tumours. Patients with positive IFCCs are prone to peritoneal metastasis and have a poor prognosis. HIPEC makes use of the synergistic effect of chemotherapeutic drugs and thermal effects to directly kill IFCCs [17, 18]. For patients with LAMN, prophylactic CRS + HIPEC treatment can achieve the greatest survival benefit, but there is an unknown risk of overtreatment. However, systemic chemotherapy before CRS + HIPEC treatment has been shown to significantly improve the prognosis of patients with peritoneal mucinous cancer [19].

Therefore, the evaluation of the prognosis of appendix mucinous adenocarcinoma mainly depends on whether the tumour is in an advanced stage, the degree of malignancy, and whether a peritoneal pseudomyxoma is formed. The spread of mucus beyond the right lower quadrant is an independent factor affecting the poor prognosis of the disease. The formation of peritoneal pseudomyxoma and the morphological evidence of suspected infiltration around the abdominal organs all indicate a poor prognosis. If the patient has mild clinical symptoms and no obvious infiltration or spread of surrounding tissue, complete removal of the appendix tissue can significantly prolong the survival period.

In summary, the case is of great significance for the indication of right hemicolectomy after appendectomy, and it also has reference value for the treatment of postoperative relapse or residual tumor.

## Data Availability

The datasets generated and analyzed during the current study are available from the corresponding author on reasonable request.

## Abbreviations

HIPEC: Hyperthermic introperitoneal Chemotherapy
CRC: Colorectal cancer
CRS: Complete cytoreductive surgery
IFCCs: Intraperitoneal free cancer cells
LAMN: Low-Grade Appendical Mucinous Neoplasms

## References

[1] Deng K, Zhang CQ, Wang GL (2014). Primary appendiceal mucinous adenocarcinoma mimicking bladder carcinoma: a case report and review of the literature [J]. Oncol Lett.

[2] Mihaela-Cristina A, Roxana C, Mădălina P (2019). Silent and surprising pathology: appendix tumors-incidental finding of a rare mucinous appendiceal adenocarcinoma [J]. Med Interna.

[3] Sehagal P, Vijay A, Sharma K (2018). A rare case of primary mucinous adenocarcinoma of the appendix presenting as ovarian mass: a diagnostic challenge [J]. J SAFOG.

[4] Tran TAN, Holloway RW, Finkler NJ (2008). Metastatic appendiceal mucinous adenocarcinoma to well-differentiated diffuse mesothelioma of the peritoneal cavity: a mimicker of florid mesothelial hyperplasia in association with neoplasms [J]. Int J Gynecol Pathol.

[5] Turaga KK, Pappas S, MPH (2013). Right Hemicolectomy for mucinous adenocarcinoma of the appendix: just right or too much? [J]. Ann Surg Oncol.

[6] Zagrodnik DF, Rose DM (2003). Mucinous cystadenoma of the appendix: diagnosis, surgical management, and follow-up [J]. Curr Surg.

[7] Karakaya K, Barut F, Emre AU (2008). Appendiceal mucocele: case reports and review of current literature. World J Gastroenterol.

[8] Agrusa A, Romano G, Galia M (2016). Appendiceal mucinous neoplasms: an uncertain nosological entity. Report of a case [J]. G Chir.

[9] Chen JX, Tang XD, Xiang DB (2012). TNM stages and prognostic features of colorectal and mucinous adenocarcinoma patients: a meta analysis [J]. Asian Pac J Cancer Prev.

[10] Basu S, Shet T (2011). FDG avid \“abdominal band\” representing omental cake in mucinous adenocarcinoma of the appendix: potential implications for disease monitoring with FDG-PET in this setting [J]. J Cancer Res Ther.

[11] Ceulemans G, Keyaerts M, Willems S (2013). Port-site metastasis after explorative laparoscopy for an incidental appendiceal mucinous cystadenocarcinoma detected with FDG PET/CT [J]. JBR-BTR.

[12] Low RN, Barone RM, Lee MJ (2013). Surveillance MR imaging is superior to serum tumor markers for detecting early tumor recurrence in patients with appendiceal cancer treated with surgical cytoreduction and HIPEC [J]. Ann Surg Oncol.

[13] Sugarbaker PH (2009). Epithelial appendiceal neoplasms [J]. Cancer J.

[14] Sugarbaker PH (1995). Patient selection and treatment of peritoneal carcinomatosis from colorectal and appendiceal cancer [J]. World J Surg.

[15] Filho A, De JG, De LEF (2011). Mucocele of the appendix: appendectomy or colectomy? [J]. J Coloproctol.

[16] Purvanov P (1997). Primary malignant appendiceal tumors [J]. Khirurgiia.

[17] Ribeiro U, Gama-Rodrigues JJ, Bitelman B (1998). Value of peritoneal lavage cytology during laparoscopic staging of patients with gastric carcinoma [J]. Surg Laparosc Endosc Percutan Tech.

[18] Ji ZH, Peng KW, Li Y (2016). Intraperitoneal free cancer cells in gastric cancer: pathology of peritoneal carcinomatosis and rationale for intraperitoneal chemotherapy/hyperthermic intraperitoneal chemotherapy in gastric cancer [J]. Transl Gastroenterol Hepatol.

[19] Milovanov V, Sardi A, Ledakis P (2015). Systemic chemotherapy (SC) before cytoreductive surgery and hyperthermic intraperitoneal chemotherapy (CRS/HIPEC) in patients with peritoneal mucinous carcinomatosis of appendiceal origin (PMCA) [J]. Eur J Surg Oncol.

